# Application of acceptance and commitment therapy in cancer-related fatigue management: insights from clinical trials and future perspectives

**DOI:** 10.1097/JS9.0000000000002305

**Published:** 2025-02-10

**Authors:** Xiaoyan Chen, Zhi Li

**Affiliations:** a Medical College of Nanchang Institute of Technology, Nanchang, Jiangxi Province, China; b Interventional Cancer Institute of Chinese Integrative Medicine, Putuo Hospital, Shanghai University of Traditional Chinese Medicine, Shanghai, China; c Hubei Provincial Clinical Research Center for Umbilical Cord Blood Hematopoietic Stem Cells, Taihe Hospital, Hubei University of Medicine, Shiyan, China

Cancer-related fatigue (CRF) is a prevalent and troubling symptom in cancer patients, with prevalence rates above 70% in individuals with advanced illness, considerably affecting daily functioning and quality of life^[1]^. Fatigue presents as severe physical tiredness, along with emotional discomfort and cognitive impairment, obstructing patients from maintaining normal lives during therapy. The processes producing CRF remain incompletely elucidated; however, they are likely linked to inflammation, metabolic dysregulation, neuroendocrine responses, and immunological abnormalities. Present treatment modalities encompass pharmacological and non-pharmacological therapy; yet, owing to the multifactorial characteristics of CRF, results frequently remain poor, and standardized management protocols are absent^[2]^. Psychological therapies, especially acceptance and commitment therapy (ACT), are increasingly recognized in supportive cancer care^[3]^. ACT focuses on improving psychological flexibility, enabling patients to accept negative emotions and physical symptoms while committing to meaningful activities. The fundamental premise advocates for the acceptance of negative emotions and physical symptoms instead of resistance, enabling individuals to participate in meaningful activities^[4]^. Unlike conventional therapies that aim to alleviate symptoms directly, ACT emphasizes altering patients’ responses to these symptoms, thereby mitigating their effects indirectly. This approach not only facilitates effective fatigue management during treatment but also sustains long-term benefits, ultimately improving patients’ quality of life. Consequently, ACT demonstrates potential in the management of cancer-related fatigue in preliminary investigations.

## Evidence from the clinical trial

In the *Journal of Clinical Oncology*, Catherine *et al*^[5]^ published a clinical trial recently that explores the use of ACT in treating fatigue in patients with metastatic breast cancer. This study was structured as a randomized controlled trial, comprising participants undergoing chemotherapy or radiotherapy, with the primary aim of evaluating the effect of ACT on fatigue and its enhancement of patients’ overall quality of life. The ACT method included training in psychological flexibility, value clarity, and commitment behaviors to aid patients in effectively managing fatigue and related symptoms. The findings indicated substantial enhancements in CRF ratings, emotional well-being, and quality of life for patients in the ACT treatment group, exhibiting more efficacy in alleviating fatigue and improving life satisfaction. The study highlighted ACT’s capacity to help patients reevaluate life values, focus on positive experiences, and enhance psychological resilience. Beyond short-term symptom relief, ACT fostered enduring coping strategies by improving patients’ psychological flexibility. This comprehensive approach underscores ACT’s relevance across diverse cancer stages and treatment phases, providing a robust theoretical and practical framework for CRF management.

## The effects of ACT

Emotional disorders in cancer patients significantly impact their quality of life and may even pose life-threatening risks. These disorders, often manifesting as anxiety, depression, fear, and other negative emotions, persist throughout the stages of cancer diagnosis, treatment, rehabilitation, and end-of-life care. Implementing targeted psychological interventions to address emotional disorders in cancer patients is crucial for improving their quality of life. The pathophysiology of CRF is complex, involving cancer therapies, immune responses, inflammation, and cognitive factors, which present significant challenges to conventional biological treatments^[5]^. Acceptance plays a pivotal role in ACT by helping patients acknowledge and tolerate unpleasant emotions, such as fatigue, emotional distress, and physical discomfort, without attempting to suppress or avoid them. This process alleviates the psychological burden associated with CRF and fosters greater emotional resilience. On the other hand, commitment serves as a core strategy within ACT, assisting patients in detaching from distressing or unhelpful thoughts. By shifting attention from the content of these thoughts to the way they are experienced, cognitive defusion reduces their influence, thereby mitigating their impact on patients’ behavior and overall well-being^[6]^. Additionally, ACT employs mindfulness-based techniques to enhance present-moment awareness, helping patients focus on the here and now. This approach reduces rumination and worry related to cancer uncertainties or future outcomes. Improved awareness enables patients to better recognize patterns of fatigue and identify activities aligned with their values and goals. ACT also motivates patients to engage in meaningful activities despite experiencing fatigue. By focusing on deeply held personal values, such as spending time with loved ones or maintaining independence, patients are empowered to prioritize actions that enhance their quality of life.

Emerging research suggests that ACT may influence neurobiological pathways involved in fatigue regulation, such as reducing hyperactivity of the hypothalamic-pituitary-adrenal axis and modulating inflammatory responses^[7]^. However, the exact biological mechanisms underlying these effects remain inadequately explored, highlighting the need for further research to elucidate the molecular interplay between ACT and CRF. By addressing the cognitive, emotional, and behavioral dimensions of CRF, ACT provides a comprehensive framework for enhancing patients’ ability to cope with fatigue. Future studies should aim to refine these mechanisms, particularly by exploring their integration with pharmacological treatments, physical rehabilitation, and other complementary therapies to optimize ACT’s effectiveness across diverse patient populations. Numerous studies have demonstrated ACT’s efficacy in alleviating emotional distress, including anxiety and depression, which are frequently associated with CRF^[8]^. By promoting balanced perspectives and adaptive coping strategies, ACT enables patients to navigate life’s challenges more effectively, sustaining improvements in their quality of life over time. Key clinical trials evaluating the efficacy of ACT in managing CRF are summarized in Table 1, highlighting its potential for broad application and further development in clinical practice.

**Table 1:** Clinical trials on acceptance and commitment therapy for managing fatigue

Intervention/treatment methods	Interventional model	Masking	Enrollment	Diseases	Current status	Clinical trial
Biweekly individual ACT sessions with a psychologist (10) and a physician (3), respectively	Single group assignment	None (open label)	40	Myalgic encephalomyelitis/chronic fatigue syndrome	Completed	NCT03562325
ACT; micro breaks in everyday life	Randomized parallel assignment	None (open label)	90	Chronic fatigue syndrome/myalgic encephalomyelitis	Ongoing	NCT05168124
ACT; usual care	Randomized parallel assignment	Single (outcomes assessor)	160	Advanced lung cancer	Completed	NCT04869267
ACT plus health education; health education	Randomized parallel assignment	Single (outcomes assessor)	80	Advanced lung cancer and caregiver burden	Ongoing	NCT05885984
Exercise intervention; acceptance and commitment therapy; dietary supplement: probiotic	Non-randomized parallel assignment	None (open label)	250	Inflammatory bowel disease	Ongoing	NCT05906043
Medical hypnosis; internet-based acceptance and commitment therapy; mindfulness session; treatment as usual	Randomized controlled trial	Double (investigator, outcomes assessor)	203	Breast cancer	Completed	NCT04518085
ACT; Education/Support	Randomized parallel assignment	Single (outcomes assessor)	84	Advanced gastrointestinal cancer	Completed	NCT04010227
ACT; Education/Support	Randomized parallel assignment	Single (outcomes assessor)	250	Metastatic breast cancer	Completed	NCT03998618
ACT; Education/Support	Randomized parallel assignment	Single (outcomes assessor)	488	Advanced gastrointestinal cancer	Ongoing	NCT06532877
Cognitive behavioral therapy; ACT; mindfulness-based cognitive therapy; mindfulness-based stress reduction; meaning-centered psychotherapy; cognitive behavioral therapy for insomnia, and other cancer-related physical symptoms like pain, fatigue, and nausea	Non-randomized parallel assignment	None (open label)	115	Cancer (unknown)	Ongoing	NCT05674357

## Challenges and future directions

Despite the significant potential of ACT in managing CRF, its widespread application faces several challenges. First, resource constraints pose a major obstacle, particularly in low-income and resource-limited settings, where the lack of professionally trained therapists and necessary infrastructure hampers implementation. Second, low patient adherence and engagement remain critical issues, especially for patients with advanced disease who may struggle to commit to therapy due to physical and psychological burdens. Strategies such as simplifying therapy protocols, increasing flexibility, or introducing motivational mechanisms could help address this challenge. Furthermore, while digital ACT offers the potential to expand accessibility, issues such as data privacy, patients’ digital literacy, and the sustainability of therapeutic effects in remote settings present significant hurdles. Additionally, underdeveloped regions often face barriers related to limited internet access and device availability. Cultural and socioeconomic differences also complicate ACT’s application, as value-based interventions must align with patients’ priorities and beliefs in diverse cultural contexts. Integrating ACT with existing treatments presents practical difficulties as well, including coordination with pharmacological therapy, physical rehabilitation, and other psychological interventions. The lack of multidisciplinary collaboration and standardized guidelines further limits its broader adoption. Finally, existing research remains insufficient in providing robust evidence, particularly regarding ACT’s long-term efficacy, potential adverse effects, and mechanisms of action across different cancer types and disease stages. Addressing these challenges will require collaborative efforts to develop resource-efficient and culturally adaptive strategies, enhance multidisciplinary integration, and promote technological innovation, ultimately ensuring that ACT becomes a more accessible and effective intervention for CRF management.

The trial designs varied, but ACT has shown significant effects in improving emotional disorders, adaptive functioning, and quality of life in cancer patients, making it worthy of clinical promotion and application^[9]^. However, different results have been observed regarding the improvement of cancer-related symptoms. These variations may stem from differences in symptom severity among patients with different types of cancer, baseline levels of participants, and ACT’s non-symptom-focused approach, which might lead to less noticeable improvements in symptom management. The standardized implementation of ACT for cancer patients requires further clinical evidence for validation. Future research on ACT in the management of CRF should prioritize conducting large-scale randomized controlled trials and longitudinal cohort studies to establish its long-term efficacy and safety across diverse patient populations, including different cancer types, stages, and treatment modalities. A deeper exploration of ACT’s effectiveness in specific cancer subtypes, such as breast, lung, and colorectal cancer, is critical to understanding its broader applicability. Tailoring ACT interventions to individual patient characteristics – such as psychological states, disease progression, treatment phases, and cultural or socio-demographic backgrounds – should be a central focus. This could involve integrating psychological assessments, treatment history, and patient preferences into the development of personalized ACT protocols.

Moreover, the potential for ACT to synergize with other therapeutic approaches, including pharmacological interventions, physical exercise, and mindfulness-based therapies, warrants comprehensive investigation. Combining these strategies may optimize outcomes by addressing the multifactorial nature of CRF through complementary mechanisms. For instance, integrating ACT with anti-inflammatory drugs or exercise programs could simultaneously target both psychological and physiological contributors to CRF. The digitalization of ACT presents opportunities for enhancing accessibility, particularly in resource-limited settings. However, future studies must rigorously assess the technical, ethical, and patient acceptability challenges associated with mobile applications, online platforms, or virtual reality therapies. Research should evaluate the feasibility of these methods in replicating the depth and effectiveness of traditional in-person therapy while addressing concerns such as data privacy, digital literacy, and patient-therapist interaction quality. Finally, addressing the limitations of ACT in resource-constrained environments is imperative. Developing streamlined, cost-effective, and culturally adaptable ACT protocols, along with scalable training programs for health care providers, could facilitate wider adoption. Additionally, exploring the neurobiological and immunological mechanisms underlying ACT’s impact on CRF could provide a stronger scientific foundation, fostering interdisciplinary collaborations and innovative therapeutic designs. By addressing these challenges and opportunities, ACT has the potential to evolve into a more effective, accessible, and sustainable therapeutic option for cancer-related fatigue management.

## Conclusion

CRF is a significant clinical challenge, highlighting the need for innovative interventions like ACT. By addressing the psychological and behavioral aspects of fatigue, ACT provides a comprehensive approach to CRF management. To enable its broader adoption, future efforts should refine protocols, incorporate digital tools, and integrate ACT into multimodal care, ensuring improved quality of life for cancer patients globally.

## Data Availability

Not applicable.
